# Corrigendum: Hydrogel microphones for stealthy underwater listening

**DOI:** 10.1038/ncomms13114

**Published:** 2016-09-28

**Authors:** Yang Gao, Jingfeng Song, Shumin Li, Christian Elowsky, You Zhou, Stephen Ducharme, Yong Mei Chen, Qin Zhou, Li Tan

Nature Communications 7 Article number: 1231610.1038/ncomms12316 (2016); Published 08 24 2016; Updated 09 28 2016.

The financial support for this Article was not fully acknowledged. The Acknowledgements should have included the following: Y.M.C. gratefully acknowledges the financial support from International Science and Technology Cooperation Program supported by National Natural Science Foundation of China (No. 11674263).

