# Supplementary material for: Hydrogel microphones for stealthy underwater listening
Source: Nat Commun. 2016 Aug 24;7:12316. doi: 10.1038/ncomms12316 (PMC4999501; doi:10.1038/ncomms12316)
Supplement: Supplementary Information — Supplementary Figures 1-9, Supplementary Table 1, Supplementary Notes 1-5 and Supplementary References [file ncomms12316-s1.pdf]

Supplementary Figures

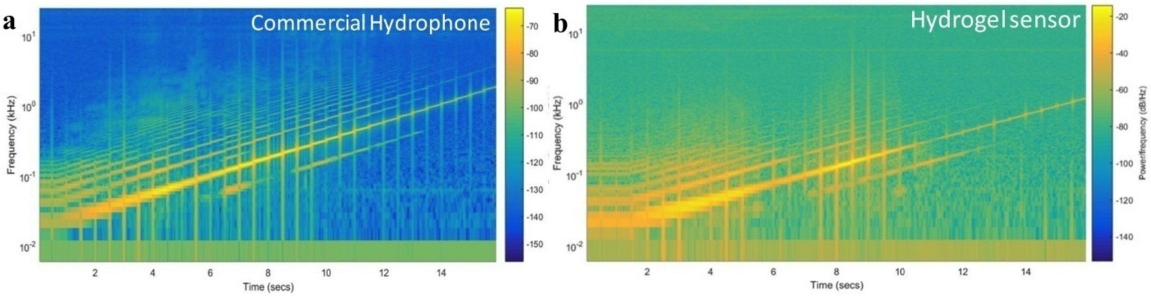

Supplementary Figure 1. Spectrogram of (a) the commercial hydrophone and (b) our hydrogel sensor. First note the high similarity between the two spectrograms, which supports our performance claim. The prominent main sweeping lines from bottom left corner to the upper right corner is shown. There are other features that indicate distortion. However, none of these features are from our hydrogel sensor. The vertical lines are artifacts due to oscilloscope not able to save ~15 s scan in one file. It divided the whole scan into 32 files, and between each file some data is lost. Therefore, there is a discontinuity (similar to a step function) of saved signal, and the Fourier transform of that is a wideband vertical line seen on the spectrogram. There are 31 lines equally spaced. The second feature is the harmonics (i.e. paralell lines in addition to the major frequency sweeping line). Since it also appears in the commercial hydrophone measurement in exactly the same pattern, these signals come from the sound generator. Currently a commercial dynamic speaker is pushed against a plastic water tank for the underwater acoustic test, which makes the coupling between the two non-ideal. Overall, none of these problems are from hydrogel sensor itself and this proves the hydrogel device is truly an outstanding underwater acoustic detector.

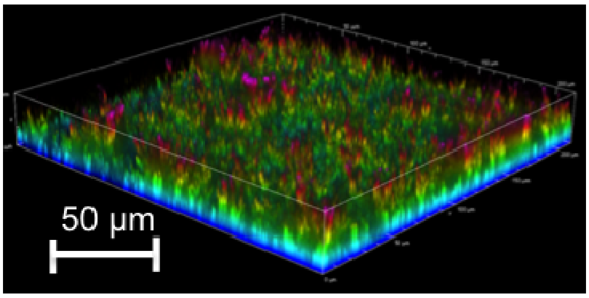

Supplementary Figure 2. Confocal laser scanning electron microscopy image of metal nanoparticles (MNP)-implanted hydrogel shows 3D distribution of those dendritic networks inside the hydrogel matrix. Due to resolution limit, details of individual network please refer to Figure 1B or Figure 2C in the main text.

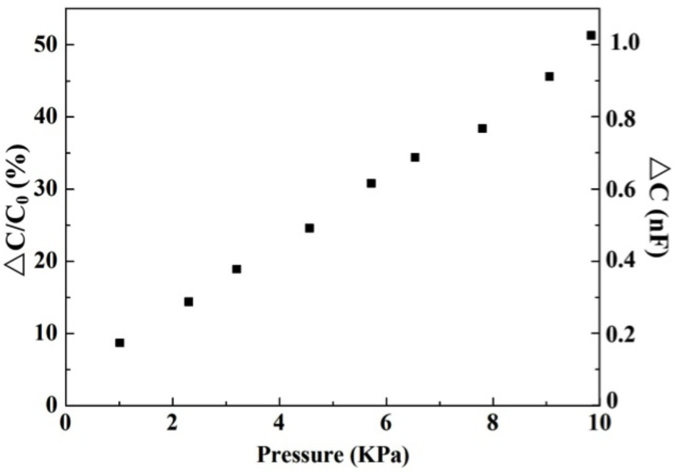

Supplementary Figure 3. The static response of the hydrogel device to external loads from 1.0 to 8.0 kPa. The device response can be quantified in two ways, one by relative capacitance change (*ΔC/C$_0$*; dimensionless) and the other by the absolute capacitance change (*ΔC*; unit of nF). While the relative response is what is usually reported in literature[1-6] it is more useful to look at the absolute response, the change in capacitance, since it determines the magnitude of the measured charge. In either case, the slope in Supplementary Figure 3 gives us an absolute device sensitivity of 0.1 nF/kPa, or a relative sensitivity of 4.8 kPa^{-1}. In comparison, a dielectric capacitor coupled with a transistor[5] has an absolute sensitivity of 15.4 pF/kPa (or 8.4 kPa^{-1}), while a recently reported dielectric device (embedded with Ag nanowires)[1] has an absolute sensitivity of 34.2 pF/kPa (or 3.8 kPa^{-1}). Clearly, the ion-rich device has far higher absolute sensitivity, and comparable relative sensitivity, in comparison to conventional dielectric sensors, mainly due to the intrinsically large electric double layer (EDL) capacitance and the deformation of MNP electrode. It is, however, worthwhile to note that the MNP networks in the hydrogel capacitor are also electrically biased (20 mV from the LCR meter), so that deformation of them can also attract or repel additional ions from the neighboring hydrogel. As a consequence, ion concentrations next to these MNP can be modulated through the combination of deformation and applied bias.

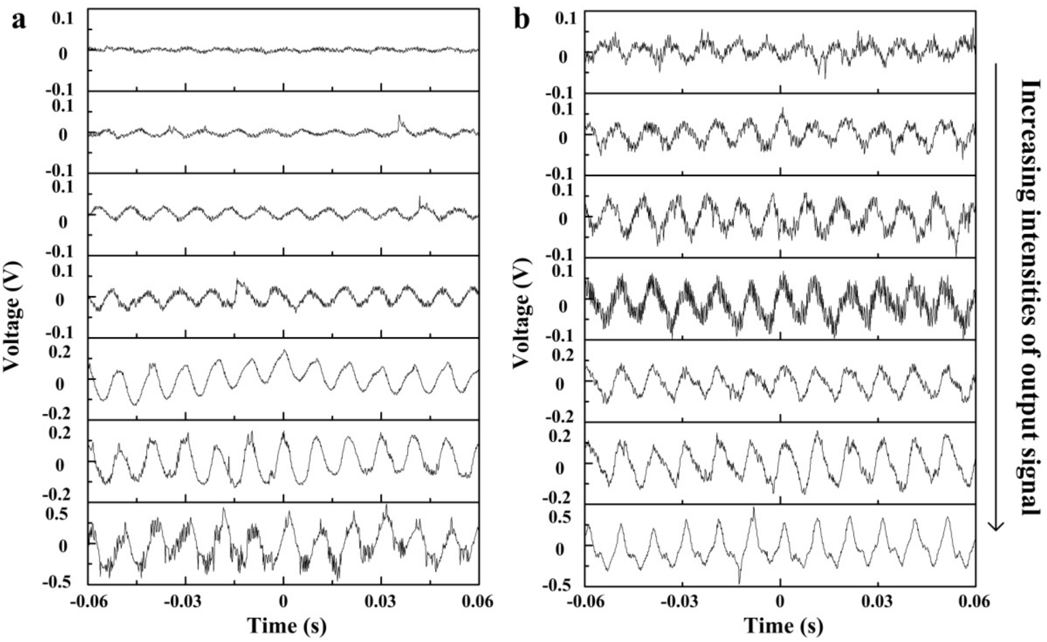

Supplementary Figure 4. Response (voltage output) of hydrogel microphones with different ion concentration (a) 10^{-2} and (b) 100 mM towards acoustic waves as in Supplementary Figure 5(c-d).

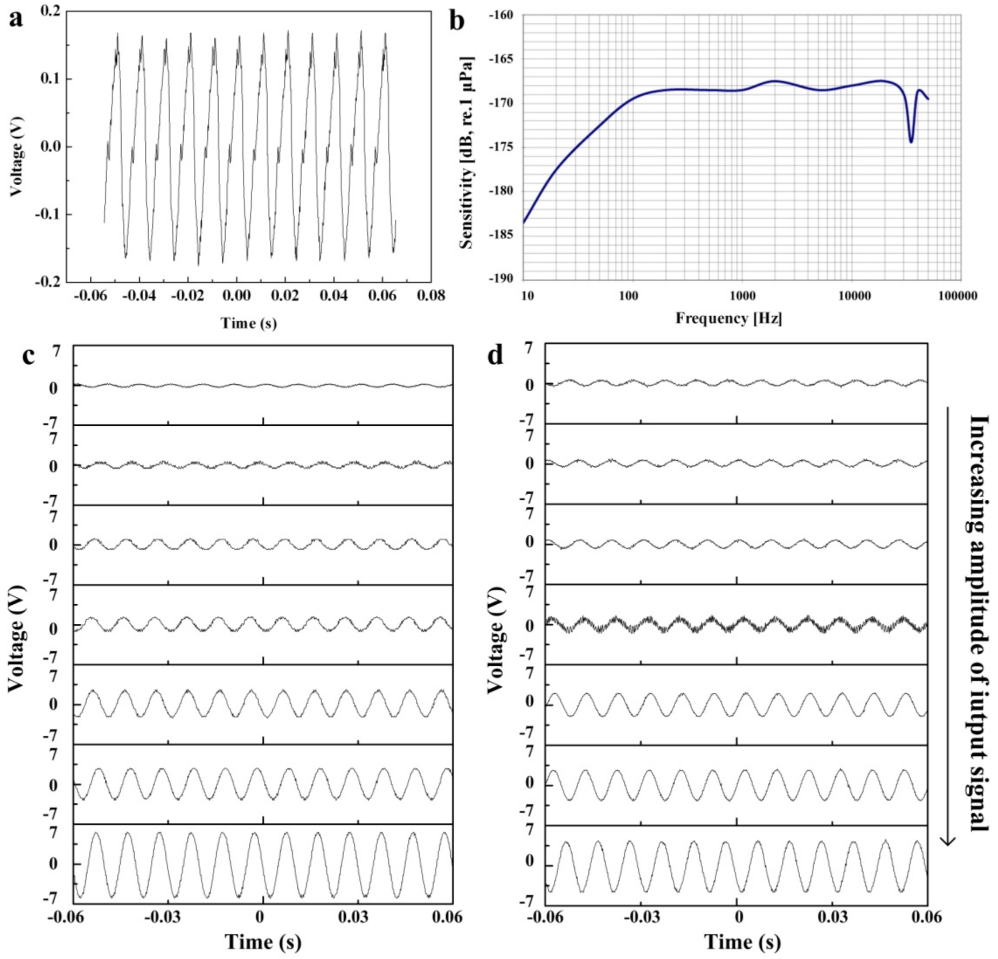

Supplementary Figure 5. (a) Response (voltage output) of a commercial hydrophone (SQ 26-07) to a selected reference acoustic wave. (b) Sensitivity map of hydrophone SQ 26-07 (provided by Cetacean Research Technology) towards varieties of frequencies (10-60 kHz). A series of acoustic waves as input signal applied on the hydrogel microphones with different ion concentrations (c) 10^{-2} and (d) 100 mM, which are recorded by oscilloscope directly. Note: These pulsed signals are from the loudspeaker/amplifier directly, not from the hydrogel sensors.

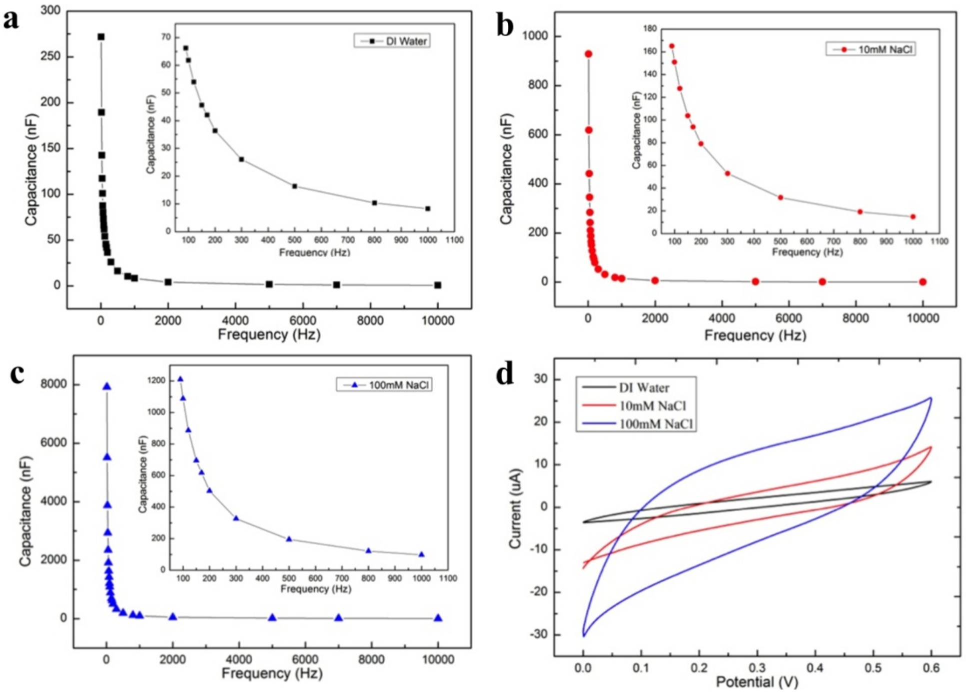

Supplementary Figure 6. (a)-(c) Capacitances of MNP-hydrogels with salt concentrations of 10^{-2}, 10, and 100 mM show a flat response only when the measurement frequency is above 1 kHz. (d) Cyclic voltammetry (10 mV/s scan) gives these samples a capacitance value of 243, 560, and 1293 μF respectively.

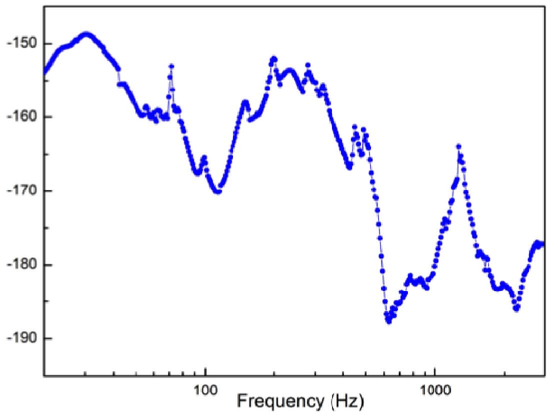

Supplementary Figure 7. Performance of hydrogel microphone with a salt concentration of 600 mM (salt concentration in ocean). Generally, at a low frequency of 20 Hz, the signal intensity is around -150 dB, but at the high frequency side (3 kHz), the noise level is around -185 dB, with both of these value slightly better than 100 mM signal in Figure 4D.

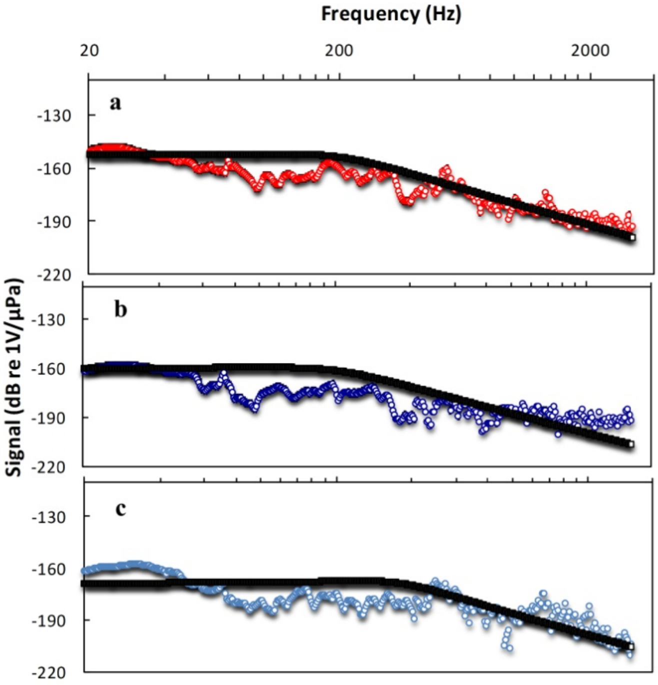

Supplementary Figure 8. Vibrational model (black markers) fitted all three MNP-hydrogel microphones used in Figure 4D. As damping coefficient is significantly large (0.5), the hydrogel network can be assumed viscoelastic.

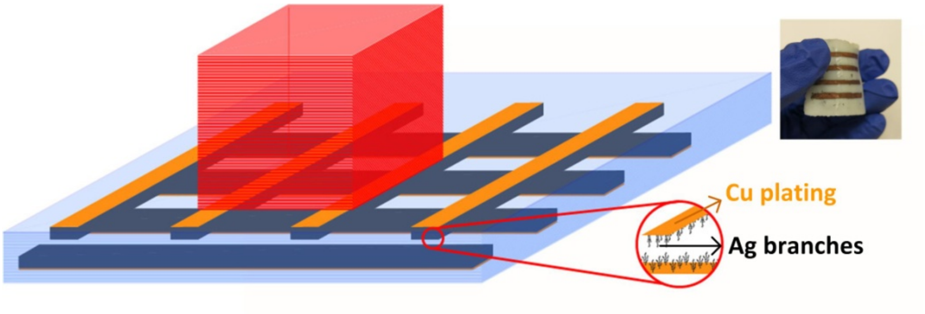

Supplementary Figure 9. Schematic of the pressure mapping test with a 4 × 4 sensing array. Inset shows the hydrogel sample used for this experiment. Briefly, a solid weight (PDMS block) with a controlled mass and bottom area is placed on a predefined location. Capacitance readings from different paired electrodes (one from the top and another from the bottom) are then recorded to map out the pressure zone on top of the hydrogel sensor array.

Supplementary Table 1. Statistic measurements of hydrogel capacitors

			Salt concentration (mM)				
			10^{-2}	1	10	100	1000
**MNP-free hydrogel**	**C_0 (nF)**	**C_{01}**	0.94	6.38	15	81.5	312.5
		**C_{02}**	0.75	7.22	17.27	90.82	365.6
		**C_{03}**	0.88	5.87	14.32	70.02	284.7
		Average	0.86	6.49	15.53	80.78	320.9
		Standard Deviations	0.097	0.682	1.545	10.419	41.104
		Relative Standard Deviations (Errors)	0.113	0.105	0.099	0.129	0.128
	**ΔC (nF)**	**ΔC_1**	0.052	0.309	0.612	2.11	5.142
		**ΔC_2**	0.04	0.361	0.732	2.51	6.11
		**ΔC_3**	0.05	0.278	0.57	1.95	4.63
		Average	0.047	0.316	0.638	2.19	5.294
		Standard Deviations	0.006	0.042	0.084	0.288	0.752
		Relative Standard Deviations (Errors)	0.136	0.133	0.132	0.131	0.142
	**$\Delta C/C_0$**	**$\Delta C_1/C_0$**	0.055	0.048	0.041	0.026	0.016
		**$\Delta C_2/C_0$**	0.053	0.05	0.042	0.028	0.017
		**$\Delta C_3/C_0$**	0.057	0.047	0.039	0.028	0.016
		Average	0.055	0.048	0.041	0.027	0.016
		Standard Deviations	0.002	0.0013	0.0013	0.0011	0.0002
		Relative Standard Deviations (Errors)	0.036	0.027	0.032	0.039	0.0137
**MNP-hydrogel**	**C_0 (nF)**	**C_{01}**	3.4	15.63	36.67	170.73	476.2
		**C_{02}**	2.3	19.33	28.84	212.18	395.11
		**C_{03}**	3.7	12.44	44.02	141.24	562.87
		Average	3.13	15.8	36.51	174.72	478.06
		Standard Deviations	0.737	3.448	7.591	35.638	83.895
		Relative Standard Deviations (Errors)	0.235	0.218	0.208	0.204	0.175
	**ΔC (nF)**	**ΔC_1**	0.83	2.52	4.44	14.39	10.11
		**ΔC_2**	0.52	3.31	3.71	16.75	8.81
		**ΔC_3**	0.85	2.11	5.68	10.97	13.07
		Average	0.733	2.646	4.61	14.04	10.66
		Standard Deviations	0.185	0.61	0.996	2.906	2.184
		Relative Standard Deviations (Errors)	0.252	0.231	0.216	0.207	0.205

		**$\Delta C_1/C_0$**	0.244	0.161	0.121	0.084	0.021
		**$\Delta C_2/C_0$**	0.226	0.171	0.128	0.079	0.022
		**$\Delta C_3/C_0$**	0.23	0.17	0.129	0.078	0.023
	**$\Delta C/C_0$**	Average	0.233	0.167	0.126	0.08	0.022
		Standard Deviations	0.009	0.005	0.0045	0.0035	0.001
		Relative Standard Deviations (Errors)	0.041	0.033	0.036	0.044	0.045

Note: Relative Standard Deviations = Standard Deviations/Average

Supplementary Note 1: Relative Electrode Area Change in Network of MNP

Our MNP-hydrogel capacitor (Cu/Ag MNP/hydrogel/ITO) adopts ITO as one of the electrodes just like the MNP-free device (Al/hydrogel/ITO), but a different counter electrode design. We consider at the ITO side (15 × 15 mm^2) the EDL capacitance has a value of C. In our control sample (Al/hydrogel/ITO), a pure aluminum foil (3 × 3 mm^2) is the counter electrode, therefore the EDL capacitance there is $C/25$. Now, a porous electrode of MNP that is m times larger in surface area replaces Al, resulting in a capacitance of $mC/25$. Capacitance (C_0) of the MNP-hydrogel device is then:

$$C_0 = \frac{C \times \frac{m}{25} C}{C + \frac{m}{25} C} = \left(\frac{m}{1+\frac{m}{25}}\right)\left(\frac{C}{25}\right) \tag{1}$$

Under pressure, the change in ion concentration (Δn_∞) in the hydrogel membrane will change the EDL capacitance in ITO side to C'. Meanwhile, the surface area in the porous MNP electrode can change, with an area now q times (assume $q = m + \Delta m$) that of an aluminum foil (3 × 3 mm^2). Capacitance change in the MNP-hydrogel device is,

$$\Delta \boldsymbol{C} = \left(\frac{\boldsymbol{m}}{1+\frac{\boldsymbol{m}}{25}}\right) \cdot \frac{\mathbf{1}}{\mathbf{25}} \boldsymbol{\varepsilon\varepsilon_0} \frac{\boldsymbol{A}}{\boldsymbol{\kappa}^{-1}} \left[\left(\frac{1+\frac{\boldsymbol{m}}{25}}{\boldsymbol{m}}\right)\left(\frac{\boldsymbol{q}}{1+\frac{\boldsymbol{q}}{25}}\right)\frac{\boldsymbol{\kappa}^{-1}}{\boldsymbol{\kappa}^{-1\prime}} - \mathbf{1}\right] \tag{2}$$

or

$$\Delta \boldsymbol{C} = \boldsymbol{C_0}\left[\left(\frac{1+\frac{\boldsymbol{m}}{25}}{\boldsymbol{m}}\right)\left(\frac{\boldsymbol{q}}{1+\frac{\boldsymbol{q}}{25}}\right)\sqrt{\mathbf{1} + \frac{\Delta \boldsymbol{n}_\infty}{\boldsymbol{n}_\infty}} - \mathbf{1}\right] \tag{3}$$

where A is the electrode area of the ITO plate. If we assume Δm is much smaller than 25, then Eqn. (3) becomes:

$$\Delta \boldsymbol{C} = \boldsymbol{C_0}\left[\left(\mathbf{1} + \frac{\Delta \boldsymbol{m}}{\boldsymbol{m}}\right)\sqrt{\mathbf{1} + \frac{\Delta \boldsymbol{n}_\infty}{\boldsymbol{n}_\infty}} - \mathbf{1}\right] \tag{4}$$

where $\Delta m/m$ equals to the relative area change in the porous MNP electrode. By plugging in relative capacitance change ($\Delta C/C_0$) and ion concentration variations ($\Delta n_\infty/n_\infty$), we can extract the values of relative area change as in Table 1.

Supplementary Note 2: Calculate Capacitance Change (∆C) in MNP-Hydrogel under Dynamic Load

Capacitance change (∆C) under dynamic load (underwater acoustic waves) is

calculated by converting response (voltage output) into a current (i) flowing through the resistor (R_f):

$$V_0 = R_f \times i \tag{5}$$

Where V_0 is the response (voltage output) of the hydrogel sensor that is simply treated as half of the peak-to-peak response ($V_{pp}/2$) and R_f is the applied resistance back in Figure 1C (value of 10^5 Ω). Meanwhile, $i = V\frac{\Delta C}{\Delta t}$, where V is the applied voltage on the hydrogel sensor (1.0 V) and Δt as 1/4 of a full cycle of our measured signal. At a sound input frequency of 100 Hz, our measured signal also has a frequency of 100 Hz, giving us a Δt of 2.5×10^{-3} s. Such that, $\triangle C = 1.25 \times 10^{-8} V_{pp}$ (F). For example, in Figure S4, when the hydrogel microphone of 10^{-2} mM (a) shows a response (V_{pp}) of 0.6 V, the calculated $\triangle C$ is then 7.5 nF. When it shows a response (V_{pp}) of 0.144 V, capacitance change is then 1.8 nF.

Supplementary Note 3: Calibration of Sound Pressures

We use a commercial listening device (hydrophone, SQ 26-07) with known sensitivity to calibrate the sound pressure in our experiments. First, acoustic wave at 100 Hz is fixed by setting volume of the loudspeaker and parameters on the amplifier. Then this sound is applied through a water tank and received by the SQ 26-07 hydrophone, with the voltage output from the hydrophone recorded by an oscilloscope. Since sensitivity (S) of hydrophone is giving by:

$$S = 20\, log_{10} \frac{V_{RMS}}{V_0} \tag{6}$$

where S can be found in Figure S5(b) at a specific frequency (for example, S = -169 @ 100 Hz), V_{RMS} the root-mean-square voltage signal from oscilloscope, and V_0 the reference voltage (1.0 V ref. 1 μPa). Then, V_{RMS} can be calculated from experimental data by converting the peak-to-peak voltage (V_{pp}):

$$V_{RMS} = \frac{V_{pp}}{2\sqrt{2}} \tag{7}$$

Based on the sensitivity (S) and recorded V_{RMS}, V_0 can be then calculated. Since each 1.0 V of V_0 corresponds to 1 μPa of pressure, a different V_0 will then tell us the sound pressure other than the reference level. For example, in Figure S5a when the

response of hydrophone shows a V_{pp} of 0.32 V, giving us a value of V_0 of 0.33 $\times 10^8$ V. Such that, calculated sound pressure will be 33 Pa. Now, we apply this same acoustic wave to our hydrogel sensor, plus a few other sounds at different intensities. We directly measure the voltage of these acoustic waves with an oscilloscope, as shown in Figure S5(c-d). As the acoustic wave back in Figure S5(a) delivers a peak-to-peak voltage of 5.6 V in Figure S5(c), then the other acoustic waves at other intensities can be calculated by scaling their voltage output vs. 5.6 V.

Supplementary Note 4: Vibrational Model for the MNP-hydrogel Microphone to Acoustic Waves

Response of our hydrogel microphone at different acoustic loads can be modeled as a mass, a spring, and a damper that are connected in parallel. A dynamic load such as an acoustic wave then produces a vibration of those combined elements at a giving frequency. A standard solution of resulting vibrational displacement (X) is: $(F_0/k)/\sqrt{(1-r^2)^2+(2\varsigma r)^2}$, where F_0 is the amplitude of incoming acoustic wave, k the spring constant, r the ratio of input frequency (f) vs. natural vibrational frequency (f_n) of the system, and ς the damping coefficient. Inside this solution, we can select appropriate parameters like F_0/k, f_n, and ς to fit experimental results. Generally, the shape of the curve will be defined by the last two parameters but the magnitude by the first parameter. To fit three situations in Figure 4D, both the parameters f_n and ς can be kept at the same value (f_n = 350 Hz and ς = 0.5), but with different value of F_0/k. Fits are all plotted as black curves overlapping the experimental data as showed in Supplementary Figure 7. Particularly, the top curve (100 mM) has F_0/k value of 70, the middle curve (100 mM; copper positive and ITO negative biased) a value of 48, and the bottom one (10^{-2} mM; copper negative and ITO positive biased) a value of 40. As the MNP structure and hydrogel matrix remained the same in all three situations, from these numbers, we can generally conclude that ion concentration or the biasing directions determines the value of spring constant ($1/k$), with the MNP structure and polymer network respectively for

natural frequency (f_n) and damping coefficient (ς).

Supplementary Note 5: Model for Sensor Time Delay

A time delay of ~15 ms was measured using the phase shift data of the sensor (Figure 4F). This cannot be explained by the time needed for sound propagation (distance from speaker to detector is ~10 cm, causing ~0.1 ms delay using sound velocity of ~ 1000 m/s in water). The short delay is verified with hydrophone measurement. We found that the delay is also related to the ion concentration in hydrogel. To explain the delay specific to our hydrogel microphone, a slow-propagating wave with linear dispersion (so velocity is constant) is required. This wave can be defined as *ion concentration wave*, so that when the electrically grounded side of the hydrogel sensor generates an ion concentration variation due to the incoming sound waves, a periodical disturbance of the ion concentration will propagate towards the biased side of the hydrogel, causing charge variation on the amplifier input. The model is established below:

Due to the ion mean free time scale is much shorter than our device response time scale, we can assume a constant ion velocity under external electrical field E, so that an electrical mobility μ in hydrogel is established where the drift velocity v of ion is

$$v_{drift} = \mu E \tag{8}$$

The concentration variation in the hydrogel membrane will result in a diffusion velocity:

$$v_{diffusion} = k_D \nabla n \tag{9}$$

Under a constant bias voltage at steady state, a leak current through hydrogel established a constant electric field inside the gel. However the ion velocity is zero because total ion number stays constant and ions cannot recombine at the electrodes as long as the bias voltage is smaller than the electrolysis voltage:

$$k_D \nabla n - \mu E = 0 \tag{10}$$

Here we are looking for plane wave solution with wave vector in thickness direction of the hydrogel, so we drop the vector mark and use notation $v_1 e^{i(kx-\omega t)}$, where $e^{i(kx-\omega t)}$

represents propagating plane wave. A constant bias voltage is applied along the thickness direction of the hydrogel, so E can be divided into two parts: $E_0 + E_1e^{i(kx-\omega t)}$, where E_0 is the constant bias and E_1 term is the ion concentration variation caused additional electrical field. Note v, E_1 can be complex here to denote the phase factor. Here diffusion velocity caused by the concentration gradient is treated as a constant velocity v_0 balancing drift velocity and the equation becomes:

$$v_0 + v_1e^{i(kx-\omega t)} - \mu E_0 - \mu E_1e^{i(kx-\omega t)} = 0 \quad (11)$$

or

$$v_0 = \mu E_0, \text{ and } v_1 - \mu E_1 = 0 \quad (12)$$

Here the terms with time dependence is separated with terms without time dependence. The continuity equation comes from the total ion number stays constant:

$$\frac{\partial n}{\partial t} + n\nabla \cdot v + v \cdot \nabla n = 0 \quad (13)$$

Again the ion concentration n can be decomposed into two parts: $n = n_0 + n_1e^{i(kx-\omega t)}$, where the n_0 term is the unperturbed ion concentration, and n_1 term represents the perturbed concentration (note n_1 can be complex to include the phase factor). The continuity equation then becomes:

$$n_0v_1 + (v_0-\omega/k)\, n_1 = 0 \quad (14)$$

The electrical field generated by charged ions is described by Gauss's Law:

$$-\frac{q}{\epsilon_0}n + \nabla \cdot E = 0 \quad (15)$$

Using plane wave assumption on E and n, assuming a charge neutral hydrogel body we find:

$$-\frac{q}{\epsilon_0}n_1 + ikE_1 = 0 \quad (16)$$

From (14), (15) and (16), we get the equation for n_1:

$$-\frac{q}{\epsilon_0}n_1 + i\frac{k}{\mu n_0}\left(\frac{\omega}{k} - \mu E_0\right)n_1 = 0 \quad (17)$$

or

$$k = \frac{1}{\mu E_0}\omega + i\frac{qn_0}{E_0\epsilon_0} \quad (18)$$

The second term is imaginary which describes the attenuation of the plane wave as it propagates. The first term is the propagation term, and we see that the group velocity of propagation is:

$$v_g = \frac{\partial \omega}{\partial k} = \mu E_0 \quad (19)$$

The bias field is on the order of 0.2 V over 1 mm thickness (10^2 V/m), ion mobility for sodium in water is on the order of 2×10^{-4} $m^2V^{-1}s^{-1}$. The ion concentration wave is therefore estimated to propagate with a velocity on the order of 0.04 m/s. Going across the 1 mm thickness would take ~ 25 ms, which agrees with the experiment on the order of magnitude.

Supplementary references

1. Joo, Y.*, et al.* Silver nanowire-embedded PDMS with a multiscale structure for a highly sensitive and robust flexible pressure sensor. *Nanoscale* **7**, 6208-6215 (2015).
2. Mannsfeld, S. C. B*, et al.* Highly sensitive flexible pressure sensors with microstructured rubber dielectric layers. *Nat. Mater.* **9**, 859-864 (2010).
3. Sun, J. Y., Keplinger, C,. Whitesides, G. M., Suo, Z. G. Ionic skin. *Adv. Mater.* **26**, 7608-7614 (2014).
4. Zhao, X. L., Hua, Q. L., Yu, R. M., Zhang, Y., Pan, C. F. Flexible, stretchable and wearable multifunctional sensor array as artificial electronic skin for static and dynamic strain mapping. *Adv. Electron. Mater.* **1**, 1500142 (2015).
5. Schwartz G.*, et al.* Flexible polymer transistors with high pressure sensitivity for application in electronic skin and health monitoring. *Nature Commun* **4**, 1859 (2013).
6. Wang J.*, et al.* A highly sensitive and flexible pressure sensor with electrodes and elastomeric interlayer containing silver nanowires. *Nanoscale* **7**, 2926-2932 (2015).
